# Recent Developments in the Determination of Biomarkers of Tobacco Smoke Exposure in Biological Specimens: A Review

**DOI:** 10.3390/ijerph18041768

**Published:** 2021-02-11

**Authors:** Hernâni Marques, Pedro Cruz-Vicente, Tiago Rosado, Mário Barroso, Luís A. Passarinha, Eugenia Gallardo

**Affiliations:** 1 Centro de Investigação em Ciências da Saúde (CICS-UBI), Universidade da Beira Interior, 6200-506 Covilhã, Portugal; hefm976@gmail.com (H.M.); pedromvcruz@hotmail.com (P.C.-V.); tiago.rosado@ubi.pt (T.R.); lpassarinha@fcsaude.ubi.pt (L.A.P.); 2 Laboratório de Fármaco-Toxicologia, UBIMedical, Universidade da Beira Interior, 6200-284 Covilhã, Portugal; 3 UCIBIO, Applied Molecular Biosciences Unit, Departamento de Química, Faculdade de Ciências e Tecnologia, Universidade NOVA de Lisboa, 2829-516 Caparica, Portugal; 4 C4—Centro de Competências em Cloud Computing da Universidade da Beira Interior, 6200-284 Covilhã, Portugal; 5 Serviço de Química e Toxicologia Forenses, Instituto Nacional de Medicina Legal e Ciências Forenses, Delegação do Sul, 1150-219 Lisboa, Portugal; mario.j.barroso@inmlcf.mj.pt

**Keywords:** tobacco smoke biomarkers, biological specimens, sample preparation, analytical developments

## Abstract

Environmental tobacco smoke exposure (ETS) and smoking have been described as the most prevalent factors in the development of certain diseases worldwide. According to the World Health Organization, more than 8 million people die every year due to exposure to tobacco, around 7 million due to direct ETS and the remaining due to exposure to second-hand smoke. Both active and second-hand exposure can be measured and controlled using specific biomarkers of tobacco and its derivatives, allowing the development of more efficient public health policies. Exposure to these compounds can be measured using different methods (involving for instance liquid- or gas-chromatographic procedures) in a wide range of biological specimens to estimate the type and degree of tobacco exposure. In recent years, a lot of research has been carried out using different extraction methods and different analytical equipment; this way, liquid–liquid extraction, solid-phase extraction or even miniaturized procedures have been used, followed by chromatographic analysis coupled mainly to mass spectrometric detection. Through this type of methodologies, second-hand smokers can be distinguished from active smokers, and this is also valid for e-cigarettes and vapers, among others, using their specific biomarkers. This review will focus on recent developments in the determination of tobacco smoke biomarkers, including nicotine and other tobacco alkaloids, specific nitrosamines, polycyclic aromatic hydrocarbons, etc. The methods for their detection will be discussed in detail, as well as the potential use of threshold values to distinguish between types of exposure.

## 1. Brief Introduction

Tobacco is the only hazardous product legally available that is harmful to everyone exposed to its action [1]. Despite the awareness to the risks entailed to tobacco use, there are more than 1.3 billion users worldwide and over 8 million deaths per year due to tobacco smoke (TS) [2]. However, the health issues related to TS are not limited to active smokers, but also to those exposed to passive or second-hand smoke [2]. The term “passive smoking” is associated with the involuntary inhalation of TS within the immediate surroundings, generally formed as a result of the burning of a cigarette (side-stream smoke 57–85%) or the inhalation of the smoke from a smoker (mainstream smoke 15–43%) [1]. On the other hand, the exposure to environmental tobacco smoke (ETS), also called second-hand smoke, has been associated with an increased risk of developing cardiovascular diseases [3], lung cancer, and other respiratory diseases [4]. Nowadays, more than 5000 constituents have been identified in TS, including a wide variety of inorganic substances, such as ethers, hydrocarbons, phenols, alcohols, ketones, aldehydes, carboxylic acids, and amines, including at least 60 different carcinogenic products [5]. Depending on the amount of smoke present, the type of ventilation, along with other environmental conditions and the level of exposure, many countries have developed restricted smoking policies and active strategies to decrease exposure to ETS in workplaces and homes. Thus, the central aims of this paper are (i) to summarize the current biomarkers of tobacco and their derivatives; (ii) to review their presence and identification in biological samples; and (iii) to revise the existing analytical approaches to track the consumption and exposure to tobacco. 

## 2. Biomarkers 

Typically, biomarkers can give a useful insight into environmental exposures, and in this particular case, into tobacco-related exposure [6]. The levels of these substances can be determined by measuring either the parent molecule or its metabolites in biological matrices [6]. To be considered an ideal ETS biomarker the substance should be highly sensitive, specific, have a usable biological half-life and should allow distinguishing between active and non-tobacco users [7]. The following sections summarize the most commonly-used biomarkers related to tobacco smoking and ETS from several chemical classes.

### 2.1. Nicotine and Tobacco Alkaloids

**Figure 1:**
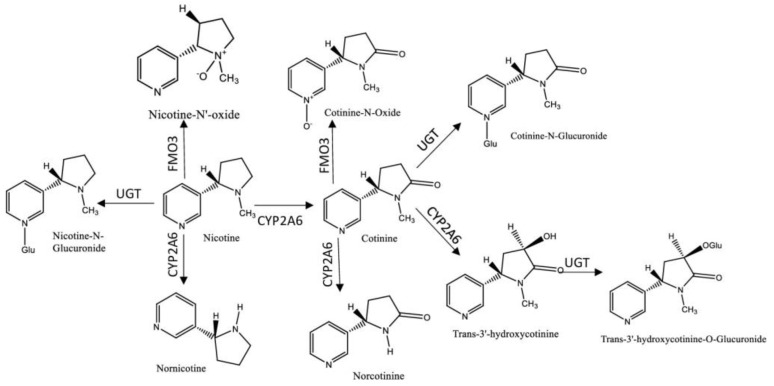
Metabolic profile of nicotine. Legend: (UGT-UPD-glucuronosyltransferases; FMO3-Flavin-containing monooxigenase 3; CYP2A6- cytochrome P450 2A6).

Nicotine is the most abundant alkaloid found in the tobacco leaf and the primary reason for tobacco dependence, due to its addictiveness [8]. Despite the existing evidence of the presence of nicotine in certain fruits and vegetables, for example, tomatoes and potatoes, the difference in the magnitude of concentrations by comparison with those in cigarette smoke or in nicotine replacement therapy (NRT) (e.g., chewing gum or nicotine patches) are much lower [9,10]. One cigarette contains in average from 7 to 24 mg of nicotine and the average nicotine air concentration ranges from 1 to 10 μg/m^3^ in indoor smoking environments, with approximately 0.3 to 3.0 mg being absorbed into the body per cigarette consumed via inhalation or absorbed through the skin [9]. Thus, nicotine was one of the first tobacco biomarkers used to access ETS, due to its high concentrations in the organism and its specificity [6]. However, its half-life is short (~2 h) and the metabolism rate is variable, limiting the time-window for its monitoring [7]. Considering all these factors, multiple nicotine metabolites started to be studied as biomarkers of ETS exposure [11]. It was reported that approximately 70/80% of the nicotine absorbed by the body was rapidly metabolized into cotinine [6] by cytochrome P450 2A6 (CYP2A6), whose values described in the literature are above 70% [12,13]. Additionally, cotinine can be further metabolized by the UGT enzymes into cotinine-*N*-glucuronide; nicotine is also converted by these enzymes into nicotine-*N*-glucuronide. Both nicotine and cotinine, as well as their glucuronides can be detected and measured in biological fluids [7]. Other compounds are formed in the metabolization process, such as *trans*-3′-hydroxycotinine, norcotinine, nornicotine, and the glucuronized compounds, among others [12,13,14,15]. Figure 1 resumes the metabolic profile of nicotine. 

Cotinine has a half-life of about 18 h in the organism and persists in biological matrices due to its poor lipid solubility, facilitating its identification in several tissues and biological fluids, including hair, blood, oral fluid, urine, and breast milk, making it an efficient and widely used biomarker [16,17,18,19,20]. Despite cotinine levels are affected by several factors, such as gender, genetic variability, pregnancy, as well as certain diseases, generally active smokers display two to three times higher concentrations of cotinine than non-smokers, allowing a simple differentiation between active- and non-smokers [21]. Although cotinine is considered an efficient biomarker to measure nicotine consumption, it does not provide information regarding the remaining metabolites. Thus, researchers usually measure the total nicotine equivalents (TNE), which is the sum of urinary nicotine, cotinine, as well as several other metabolites in the nicotine metabolic profile, to fully evaluate nicotine intake; as such, compounds as hydroxy-PAHs and 4-(methylnitrosamino)-1-(3-pyridyl)-1-butanol (NNAL) may be measured [22,23]. *Trans*-3′-hydroxycotinine is, in most individuals, the major metabolite of cotinine, making it an important biomarker to measure the ETS exposure [24]. In urine, the levels of *trans*-3′-hydroxycotinine surpass those of cotinine by about three to four fold, but in plasma and oral fluid cotinine levels are higher than the former’s, allowing the differentiation between smokers and non-smokers. [25]. Other substances, like anabansine and anatabine, minor tobacco alkaloids, are also established tobacco intake biomarkers. Additionally these compounds are not present in NRT, and therefore may be used to indicate tobacco use by individuals undergoing NRT [26]. 

### 2.2. Carbon Monoxide (CO)

CO is a product generated by incomplete combustion of organic materials during combustions, both from tobacco and non-tobacco sources, for example motor vehicles, forest fires, etc. [27]. Exposure to CO can be measured by measuring the concentration of carboxyhemoglobin (COHb), a complex formed from carbon monoxide and hemoglobin in red blood cells (percent of hemoglobin saturation) and the concentration of CO in exhaled breath (COex) [27]. Both are valid markers to identify individuals that recently used a combustible tobacco product [8]. Despite the short half-life of COHb (1 to 4 h) and COex (5 to 55 min), these biomarkers allow distinguishing between tobacco users and non-users and are considered to be the most useful biomarkers for verifying smoking cessation in clinical trials [28]. For instance, COex concentration in non-smokers ranges from 4 to 7 ppm, while levels from 20 to 30 ppm are seen in smokers (over 50 ppm in heavy smokers). Concerning COHb, non-smokers present values from 1 to 2%, smokers from 4 to 7% and heavy smokers higher 12% [27]. 

### 2.3. Tobacco-Specific N-Nitrosamines (TSNA)

**Figure 2:**
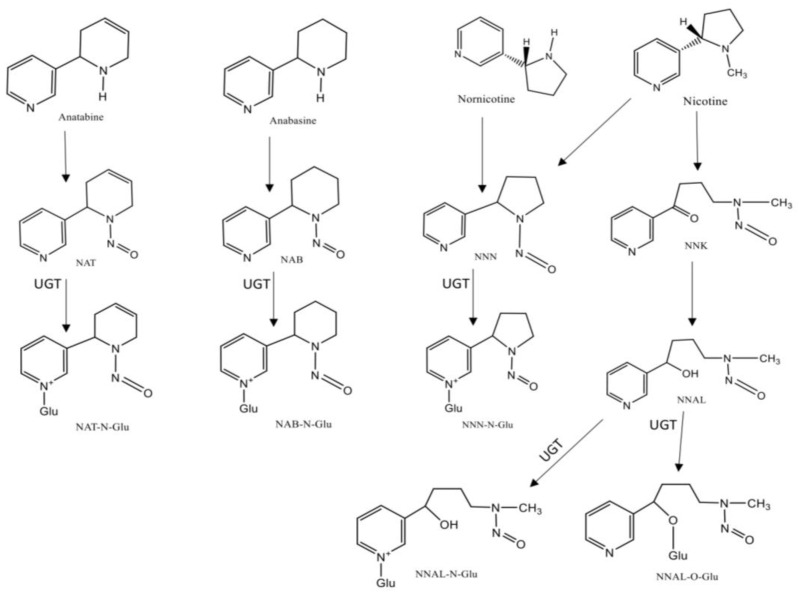
Chemical structures and metabolism of Tobacco-Specific N-Nitrosamines (TSNA). Legend: (UGT-UPD-glucuronosyltransferases).

TSNAs are formed from tobacco alkaloids during the curing process and are present in tobacco and TS, being mostly nicotine and nornicotine derivatives [29]. The most studied TSNAs are nicotine-derived nitrosamine ketone (NNK), a potent lung carcinogenic, and *N’*-nitrosonornicotine (NNN), an oral cavity and esophageal carcinogenic [30]. This class of compounds and their metabolites (Figure 2), which include NNK, NNN, NNN-glucuronide, NNAL-*N*-glucuronide, NNAL-*O*-glucuronid; the glucuronide metabolites, also referred to as NNAL-Gluc, and N′-nitrosoanabasine (NAB), NAB-glucuronide, N′-nitrosoanatabine (NAT), and NAT-glucuronide, are considered the most relevant biomarkers for ETS monitoring [29,31,32]. From these, NNAL and NNAL-Gluc, the main metabolites of NNK, are the most widely studied biomarkers of this class, displaying a long half-life in biological fluids (~10 to 45 days), they are completely tobacco specific and may be detected in urine [33,34]. In several studies ranges of 1–2 pmol NNAL/mL of urine in smokers have been reported, while in non-smokers exposed to ETS the concentrations were 1–5% of the amount found in smokers [35,36]. In addition, this biomarker is never found in non-tobacco users that are not exposed to ETS, and correlates well with other tobacco-specific markers such as cotinine and TNE [8]. Monitoring NNAL has already been used in investigations regarding exposure to NNK in non-smokers, for example in newborns, children and women living with smoking partners [37,38]. For instance, NNN and its glucuronides, can also be measured in urine and in toenails. However, due to differences in their biological pathways, these compounds exist in lower concentrations than NNAL in the organism, which can difficult their monitoring [29]. In urine, it is possible to distinguish smokers from non-smokers based on the levels of NNN, and it was also demonstrated that it correlates with increasing cigarettes per day and the total levels of cotinine found in urine [39]. 

### 2.4. Polycyclic Aromatic Hydrocarbons (PAHs)

PAHs are chemicals formed by the incomplete combustion and pyrolysis of tobacco and other organic matter, such as pyrene, fluorene, phenanthrene, and naphthalene [40]. Therefore, both non- and carcinogenic PAHs exhibit high correlation with TS [8]. PAHs biomarkers include 1-hydroxypyrene, less used due to its low specificity to ETS, tetrols of benzo[a]pyrene and phenanthrene, considered effective biomarkers of PAH uptake and metabolic activation [41]. In several studies it was demonstrated that the levels of PAHs biomarkers are higher in smokers when compared to non-smokers [42]. Specifically, the levels of benzo[a]pyrene and phenanthrene-tetrols are two to three times higher in smokers than in non-smokers [43].

### 2.5. Volatile Organic Compounds (VOCs) 

VOCs are substances that are generally formed by the incomplete combustion of organic materials, such as some components of paint, cleaning supplies, pesticides, and are also present in TS [44]. This class includes a wide variety of compounds that can be measured in biological fluids [44]. In blood, 2,5-dimethylfuran, which is comparable to serum cotinine in sensitivity and specificity, benzene, toluene, ethylbenzene, xylene, and styrene are directly correlated with active smoking and have dose-response relationships with cigarettes-per-day [45]. The main VOCs found in urine are mercapturic acids of ethylene oxide, acrolein, crotonaldehyde, butadiene, benzene, acrylonitrile, and acrylamide; these compounds are present in higher amounts in smokers that non-smokers [46]. Particularly, the concentration of 3-hydroxypropylmercapturic acid, a mercapturic acid metabolite of acrolein, is present in smokers at concentrations four times higher than those of non-smokers [47]. Most mercapturic acid metabolites of VOCs present in urine only remain for one day following smoking cessation, making them good biomarkers to distinguish between active and non-smokers [8]. 

### 2.6. Aromatic Amines and Heterocyclic Amines 

Both aromatic and heterocyclic amines are combustion products that are present in the particulate phase of TS with great potential for evaluation of the exposure to ETS [48,49]. Most of the amines identified as ETS biomarkers are found in small amounts in biological fluids, often in the low part per billion (ppb) range [49], and therefore hardly differentiate between smokers and non-smokers [8]. Indeed, urinary levels of 1-naphthylamine, 2-naphthylamine, ortho-toluidine, 3-aminobiphenyl, 4-aminobiphenyl and the majority of heterocyclic amines are generally low, with the exception of 2-amino-1,7-dimethylimidazo[4,5-*b*]pyridine (DMIP) and 2-amino-9*H*-pyrido[2,3-*b*]indole (AαC), that can be used to distinguish smokers from non-smokers [49].

### 2.7. Metals

Metals, such as lead and cadmium, may be also considered ETS biomarkers, despite being widespread in the environment, since the tobacco plant can absorb these metals from the soil [8]. Previously, arsenic was also considered a tobacco biomarker because of the use of arsenic pesticides in tobacco cultivation, but this action was discontinued recently. Cadmium exhibits elevated levels in smokers compared to non-smokers in biological fluids like blood and urine, presenting very long half-lives (urine—11 to 30 years/blood—7 to 16 years) [50,51]. Hence, urine cadmium is a biomarker of cumulative and long-term ETS. On the other hand, lead is mostly detected in blood and urine, and many studies associate exposure to this metal to increased risks to develop cardiovascular diseases [52].

### 2.8. Thiocyanates

One of the first biomarkers of exposure to ETS discovered in biological fluids was thiocyanate ion (SCN^−^); indeed, it was demonstrated that this ion was found at higher concentrations in smokers than in non-smokers [27]. This ion is a metabolite of cyanides (HCN), which are found in foods like almonds, nuts, leguminous plants, cow’s milk, etc., however in small quantities [27]. However, larger concentrations are produced by the metabolism of some constituents of TS [53]. SCN^−^ exhibits a longer half-life than most biomarkers (~6 days) and the levels of this compound in urine and serum are two to three times higher in smokers than in non-smokers [27]. The average concentration of SCN^−^ in serum and urine samples is 150 µmol/dm^3^ in smokers, compared to 50 µmol/dm^3^ in non-smokers, while in oral fluid the levels are in the order of 3000 µmol/dm^3^ in smokers and 1200 µmol/dm^3^ in non-smokers [27].

## 3. Biological Specimens and Cut-Off Concentrations

There are several biological specimens that can be used to determine biomarkers of exposure to ETS, and the main differences are related with the time window available for their determination. For clinical analysis, serum and plasma are the most traditionally used samples, since whole blood requires more manipulation steps and more time-consuming laboratory procedures [54,55]. This specimen is particularly useful, since the detection of toxic compounds strongly suggests recent exposure. On the other hand, there is a significant correlation between blood levels and the effects on the body for most chemicals. For this reason, blood samples are ideal for quantitative analysis. Conversely, urine is normally used for metabolite research, although most substances are also excreted unchanged (up to approximately 2%). It is easy and non-invasive collected, and it is generally available in large quantities and has a lower number of interferences when compared to other specimens. It has great stability when frozen, allowing long-term storage of positive samples [55,56,57]. However, it is an easily tampered-with sample, since the collection is carried out in an unattended manner for privacy reasons. Most compounds remain in urine for 2 to 5 days after consumption. The use of urine is particularly indicated for immune-enzymatic assays, allowing a rapid screening of both compounds and metabolites. The urinary levels of these molecules indicate only exposure to the substances, and do not allow concluding about the exact time of exposure or the likely physiological effects [54,55,58,59].

Another specimen that is used is hair, an outgrowth of the hair follicle that mostly consists of proteins (keratin, 65–95%), water (15–35%), lipids (1–9%), and minerals (less than 1%) [60,61]. Hair grows at a rate of 0.6 to 1.4 cm per month, depending on the type of hair and the anatomical location [61,62]. Hair has several advantages when compared to blood and urine samples. Firstly, collection can be carried out easily in a non-invasive fashion by cutting with scissors, without the need for highly specialized personnel to collect the sample. This is in stark contrast to blood, whose collection requires needles and syringes, and urine, whose collection needs to be supervised to avoid sample manipulation or adulteration, which disturbs the individual’s privacy [61]. The main advantage of this sample in comparison to blood and urine is its much wider detection window, of weeks, months or even years, comparing to a few hours or days in the case of blood or urine respectively [61]. Hair also has several disadvantages, namely, the lack of correlation between the levels in this sample and the concentrations in blood/plasma and the possibility of detecting the substances after passive exposure (e.g., smoke, vapors, or contaminated hands), instead of after active consumption [61,63,64].

Another specimen that is generally used to evaluate the exposure to toxic substances is nails [60,65,66,67,68,69]. An important difference between nails and hair is that nails grow continuously, rather than through a growth cycle. As occurs with hair, compounds incorporation into the nails can occur externally through sweat. However, it is also possible to observe external contamination from the environment [60,67,70]. Nails grow at a rate between 1.9 and 4.4 mm per month (average of 3 mm per month) [60,71].

The speed of growth of toenails is approximately 30–50% slower than that of fingernails, and therefore windows of detection can range from 8 to 14 months. It is not possible to detect smaller time windows or changes in consumption patterns in the short term by analyzing nail fragments, so this type of analysis is more suitable for documenting average consumption behavior over long periods (e.g., for monitoring abstinence). The main obstacle is the relative scarcity of disposition studies, in addition to the fact that the mechanisms of drug incorporation are not yet fully understood. Furthermore, it is difficult to compare the results of studies already carried out, due to the lack of standardized methods of testing and sampling [60,67,70].

The use of oral fluid has been increasing, not only due to the development of more sensitive and reliable instruments, but also due to the numerous advantages it presents as a biological sample [72,73]. The concentration of the analytes determined in oral fluid represents the free, non-ionized fraction, which is the pharmacologically active, fraction of the substances in blood. Sample collection is carried out in a simple and non-invasive fashion, with the advantage of being less subject to adulteration or substitution when compared to other matrices such as urine, since it can be done in a supervised manner without infringing the individual’s privacy [73,74]. On the other hand, it has the disadvantage that it is not always easy to obtain sufficient quantities for analysis. If drugs are taken by inhalation, there may be false positives regarding the correlation with blood concentrations since there may be residual amounts of the analytes in the oral cavity [72]. The detection window of the compounds in this matrix, although it can vary according to several factors, is relatively short, oscillating between 24 and 36 h for most compounds, similarly to what happens with plasma [75].

Regarding the matrices used for the assessment of substance use during pregnancy, in the case of the determination biomarker of exposure to ETS, placenta, and meconium are the most used samples.

Placenta is a temporary organ that unites the mother and the fetus, transferring oxygen and nutrients from the mother, allowing the release of carbon dioxide and waste from the fetus. Placenta is easily and non-invasively collected at the time of birth, and functions as a drug deposit, being as such useful to identify situations of intrauterine exposure [76]. Meconium is the first fecal matter passed by a newborn, normally excreted during the first 72 h of life (20 to 70 g). Its formation begins around the 12th week of gestation and it accumulates until birth. [72]. Meconium analysis can provide an overview of drug exposure during the last quarter of pregnancy. Drugs are stable in meconium for a period of up to two weeks at room temperature. The disadvantages of this sample are related to the need for sequential sampling over time, requiring up to five days, or the loss of sample, in case of excretion in the uterus. In addition, meconium can be contaminated by the newborn’s urine, resulting in positive results for drugs administered during delivery [60,72,77].

Regarding the publications, it is possible to use other biological samples such as liver [15], semen [78], or nasal fluid [79] to determine these biomarkers.

In order to distinguish active from passive smokers, statistical analysis are carried out in several studies, based on different conditions and biomarkers. In order to be able to establish consumption patterns and the metabolism of the markers to be identified, it is necessary to know the half-life of the compounds. In plasma samples, nicotine, cotinine, and *trans*-3′-hydroxycotinine have half-lives of 1.5–3, 6–22, and 5–8 h, respectively, and these data apply to the remaining samples [80]. Based on the analysis of half-life times we find that cotinine analysis as a marker of ETS is advantageous because of its longer half-life, shorter fluctuation levels, and it is a metabolite originating from nicotine metabolism in the body [13]. TSNA markers as NNAL, due to its half-life time (10–16 days) [81] are very useful to define the type of exposure and to define whether or not smoking cessation occurred. However, in order to define whether exposure to tobacco smoke has occurred actively or passively, several studies have been conducted to define the cut-off that best define an active or passive consumer. Several nicotine and cotinine cut-off concentrations have been suggested by several authors, 10 ng/mL being the most consensual [14,80,82]; in the case of hair a 5 ng/mg cut-off is used [83]. However, 2 ng/mL anatabine cut-off was described to be a reliable indicator of ETS recent exposure [84]. NNAL cut-off for active smoking was established as 14.4 pg/mL in urine [81]. In order to confirm whether ETS exposure is passive or active, the ratios between different compounds are also compared, which may be different depending on the type of sample being analysed. One of the conclusions that we can observe after analysing the ratios for plasma and oral fluid for electronic cigarettes and regular tobacco is that the values observed in oral fluid are more than 1.5 times higher than those observed in plasma, and that plasma nicotine values are lower than cotinine’s [85]. Cotinine plasma to whole blood ratios were higher than one, which indicates that the cotinine value in blood samples is influenced by the cotinine value in plasma [86]. For TSNA, the described ratios of NNN/NNAL are 2.8 and 0.1 in nails and urine respectively, which showed a strong relationship between NNN and NNAL in nails and cotinine [87]. One of the most interesting data is the fact that the ratios between NNAL and cotinine are 10 times higher for passive smokers than for active smokers, and this is an important factor to observe in making this distinction [81]. The NNK/nicotine ratio makes it possible to check the ventilation of smoking spaces, as its ratio increases with the “aging of the smoke” [81].

## 4. Sample Preparation Techniques

The most commonly procedures in analytical toxicology are pre-concentration and clean-up methods that will be chosen depending on several factors. Regardless, other biological matrices can be used as samples for the exposure to ETS, urine is one of the most common biological matrices used in the studies described in Table 1.

Several liquid-liquid extraction (LLE) studies for tobacco smoke exposure have been made with liquid matrices. Advantages of LLE come from the large body of solvent extraction literature, with versatility on choice of organic solvent, pH, type, and concentration, affecting both the selectivity required for sample clean-up and the volume necessary for preconcentration of the target analytes [105]. Perez-Ortuño et al. [89] described a urine LLE technique based on a mixture of internal standard (IS) solution with the sample and dichloromethane with recoveries over 90% with low carryover. Toraño et al. [96] determine thiocyanate, nicotine and cotinine in hair, oral fluid, and urine. However, the analysis for thiocyanate could not be performed at the same time than that of nicotine and cotinine in oral fluid or hair, because its concentration in oral fluid is high, while it is absent from hair. For this purpose hair samples were washed with dichloromethane to remove external contamination. It was added 50 µL of 4 M NaOH to 20 mg of hair in 800 µL water, 800 µL of oral fluid or urine. The hair sample was incubated at 100 °C for 10 min for digestion. Then 100 µL of dichloromethane (for oral fluid and hair samples) or 150 µL of an alkylating extraction solution (for urine samples) and 100 µL of a saturated NaCl solution were added. The solutions were shaken for 30 min, centrifuged and 2 µL of the extract was injected into the GC-MS system. The GC-MS system was chosen in order to determine all analytes in a single method; however, thiocyanate had to be quantified as a pentafluorobenzyl derivative.

Typically, and in order to establish correlation and ratio studies between plasma and oral fluid nicotine and metabolites in regular tobacco and e-cigarettes, a mixture of chloroform/isopropyl alcohol was used in the extraction process [85]. For TSNAs, nicotine and the metabolites, after hair incubation in alkaline conditions, an LLE procedure with dichloromethane and dichloromethane:isopropanol (75:25) was used, then proceeding to neutralization and alkalization, another extraction with the same LLE method and addition of hydrochloric acid in methanol obtaining good recoveries but with some matrix effect [88]. Apart from LLE, the other most common technique for extraction was solid phase extraction (SPE), which can produce cleaner extracts than LLE. Miller et al. [12] described the use of mixed mode Oasis^®^ MCX and HLB cartridges for urine and plasma with two different conditioning methods. Prior to SPE sampling, plasma was acidified with 10% aqueous trichloroacetic acid for protein precipitation (PP) and urine was acidified with 5 mM aqueous ammonium formate (pH 2.5) with extraction recoveries above 51%. For hair samples, incubation at room temperature with 1 M sodium hydroxide was made at room temperature for 1 h and extracted with Oasis^®^ MCX with good recoveries, except for cotinine-*N*-β-*D*-glucuronide [83]. Several washing procedures were tested and compared after exposure to one and four cigarettes, and also unwashed hair samples for the same tobacco exposure, obtaining as a more favorable process in washed samples for the procedure with a commercial shampoo (Herbal Essences Hello Hydration) in a proportion of 1:4 with Milli-Q water with another two Milli-Q water washes [83]. In both studies by Miller et al. [12,83] nicotine and its metabolites were quantified. Other simple SPE extraction methods for oral fluid use Clean Screen^®^ ZSDAU020 or Oasis^®^ MCX SPE columns with good recoveries [91,92,100]. Shakleya et al. [92] method use Quantisal ^TM^ device to collect oral fluid samples. For meconium, Gray et al. [93] used a small amount of sample dissolved in methanol with 0.01% formic acid (w/v); with and without β-glucuronidase, that can be used for pharmacokinetic studies or to increase the detection levels of some parent drugs, using Clean Screen^®^ ZSDAU020. In this study some matrix effects were observed, which were adjusted by deuterated internal standards. Another work studied active vs passive exposure in oral fluid between the use of nicotine patches and tobacco smokers, using Phenomenex Trace B cartridges; the levels obtained for the patches patients were similar to those of smokers [80]. For an alternative biological matrix, such as toenails, Stepanov et al. [87] reported that the sample can be digested with 1 N sodium hydroxide and two different extraction steps with SPE cartridges can be applied, with a ChemElut cartridge followed by Oasis^®^ MCX, with dichloromethane and water/methanol/ammonium hydroxide (90:5:5) as elution solvents. A normal phase extraction (NPE) with Bond-Elut Silica cartridges with ethyl acetate as elution solvent was added to improve NNN detection. Only one study used PP alone as extraction method, both for free and total nicotine and metabolites, using cold acetone as precipitation solvent because of phospholipids insolubility [84]. One of the most recent developments in extraction techniques are automated sample extraction procedures, which can drastically reduce the sample and solvents volumes and managing to decrease analysis time [97]. Bead injection lab-on-valve (BI-LOV) coupled with µSPE (micro SPE) was capable of promoting an automatic process to extract cotinine from saliva samples previously subjected to PP with acetonitrile (4 °C, 20 min) with Oasis^®^ HLB sorbent in an eight channel unit that works with piston pumps, with SPE and LC working synchronized [97]. Stationary phase of µSPE was renewed with new micro-sorbent beads at each sample injection and recoveries of 95.9% were obtained [97]. An automated on-line SPE coupled to liquid chromatography and tandem mass spectrometry (LC-MS/MS) was described by Chen et al. [99] using a 10-port switching valve and a Inertsil ODS-3 column; two solvents were used for the extraction [5% methanol (*v*/*v*) with 0.1% formic acid and 90% acetonitrile (*v*/*v*) with 0.1% formic acid] and two more solvents were used as mobile phase for analytical quantification. More than 250 samples were analyzed, using low volumes of urine (20 µL), and no carryover was observed. In-tube SPME is another example of automation, leading to an increase on precision and sensitivity. Inukai et al. [95] uses in-tube SPME with Carboxen 1006 PLOT column for hair previously washed with dichloromethane and incubated in distilled water (80 °C for 30 min) with only 1 mg of hair samples. This methodology provided high sensitivity for nicotine and cotinine detection. Katokoa et al. [98] use the same technique for saliva and urine samples but with another type of column (CP-Pora PLOT amine capillary column) and LC-MS. The study reported good recoveries for nicotine and metabolites.

Some alternative pre-concentration approaches have been described in recent years, with several advantages, allowing designing a great variety of methods and their application to different types of samples. Specifically, a different extraction method was explored by Carmella et al. [35], that uses 96 well plates supported liquid extraction (SLE) plates and SPE. True Paper 96 well plates were used to create three fractions, one without any hydrolysis step for total NNAL, the second with enzymatic hydrolysis (β-Glucuronidase) for free NNAL, and the last with alkaline hydrolysis (0.5 M NaOH) for free NNAL + + NNAL-*N*-Gluc. Isolute SLE^+^ diatomaceous earth 96-well plates and Oasis^®^ MCX 10 mg, 30 μm SPE 96-well plate were chosen for this study [35]. Free NNAL + NNAL-*N*-Gluc fraction were incubated for 30 min at 80 °C, while for total NNAL samples were incubated at 37 °C overnight. Dichloromethane and water/methanol/ammonium hydroxide (35:60:5, *v*/*v*/*v*) were used as SLE and SPE elution solvents, respectively [35]. For r-1,t-2,3,c-4-tetrahydroxy-1,2,3,4-tetrahydrophenanthrene (PheT), a PAHs, quantification, a urine sample suffers enzymatic hydrolysis made with a solution of β-glucuronidase and arylsulfatase mix with 0.5 M sodium acetate buffer (pH5), being extracted with Strata SDB-L plates, using methanol/water (1:1, *v*/*v*) as elution solvent [35]. An SLE extraction method for liver and placenta by Swortwood et al. [15] used metal spheres to be able to crush both samples, then using 0.01% formic acid in methanol for homogenization. Prior to sample loading, 0.25% ammonium hydroxide in methanol was added to increase pH and Isolute SLE was used to proceed to nicotine and metabolites quantification with dichloromethane/isopropanol (95:5, *v*/*v*) as elution solvent. This strategy lead to a method capable of reaching the cut-offs observed in other studies [15]. Liquid-liquid microextraction technique made with chloroform was explored by Gallart-Mateu et al. [90] for nicotine quantification in oral fluid by gas chromatography coupled to mass spectrometry (GC-MS) in comparison with IONSCAN-LS IMS with good recoveries (104%). Another SPME technique use headspace-SPME (HS-SPME) with gas chromatography coupled to tandem mass spectrometry (GC-MS/MS), that was tested with two sample collection methods (spitting and Salivette^®^) for unmetabolized PAH’s [103]. Good limits of detection (LOD) and limits of quantification (LOQ) were achieved for low-boiling PAH’s. One of the latest miniaturized extraction methods is dried blood spots (DBS), which uses very small sample volumes, is easy to transport at room temperature and has low costs for transportation [86]. Two different ways of obtaining cotinine from blood were used and analyzed by DBS, one from venipuncture collection and another based on finger-prick [86]. After DBS Whatman 903 Protein Saver collection of blood, methanol was used to extract the compounds using cycles of atmospheric and 20 kpsi pressures, achieving good LOQs [86]. Molecular imprinted polymers (MIPS) are amongst the most recent novelties in the field of concentration and extraction methods due to their capacity to become selective for an analyte or group of analytes using monomer polymerization, using as model the target analyte [106]. Another example of MIPS was the Xia et al. [101] method that initially use a ChemElut column loaded with urine samples and 10 M sodium hydroxide, eluted with dichloromethane, then added hydrochloric acid and centrifuged the sample, collecting the hydrochloric acid layer that was added to NNAL MIP SPE with 10 M sodium hydroxide and 0.5 M phosphate buffer (pH 6.4). The sample was then eluted through three sequences of methylene chloride and reconstituted in water [101]. For total TSNA quantification, β-glucuronidase was added, incubating for 24 h at 37 °C [101]. NNAL MIP was selected because of NNAL recoveries compared with TSNA MIP, even if the yields of the other compounds are lower. However, NNAL is the metabolite of greatest interest, as both it and NNK are heavily carcinogenic [101]. For the determination of thiocyanate, direct injection [78,79] or accelerated solvent extraction (ASE) with water and diatomaceous earth was used has extraction methods with good recoveries described by Narkowicz et al. [102].

A novel technique was reported by Hu et al. [107], who proposed a microfluidic gas collecting platform that allowed both sample extraction and analysis. In detail, an arrayed alveolar model was developed on a chip, with the possibility of being driven by a periodic pressure generation system that mimics the human respiratory motion. The connection between the array on chip and the side stream smoke (SS) generation by gas channels enables to inhale and exhale gas, after which a droplet array is assembled on the alveolar area for gas collection. The captured substances are subsequently analyzed. The main advantages of this new approach are the fact that is small, cheap, and portable. Additionally, it can be used for a wide range of applications in inhaled air pollutant analysis.

## 5. Analytical Techniques for the Determination of Biomarkers

One can understand the increasing interest in monitoring the use of tobacco products and the exposure to second hand smoke in a wide range of biological specimens. Many studies also involve the non-smoker population, hence low concentrations are expected to be found. The use of biomarkers to measure the real exposure to TS becomes, then, very important for an accurate interpretation, more reliable than using data through questionnaires [89]. It is also relevant to consider that the samples’ volume might be low, especially in alternative samples’ cases, and in order to quantify the diminished concentrations present, analytical methods with high sensitivity are usually required [6].

Immunological techniques have been extensively applied for the determination of tobacco smoke biomarkers, among them, radioimmunoassay (RIA) and enzyme linked immunoassay (ELISA) are the most popular [6]. However, these are known to give cross-reactions, hence they are not the most suitable methods for quantification. RIA is reported to result in cross-reactivity for desmethylcotinine and (-)-cotinine *N*′-oxide, while in ELISA methods, an overestimation of the cotinine concentration has been observed because of cross-reactions towards other cotinine metabolites, namely dimethylcotinine and hydroxycotinine [6]. Overall, most cotinine immunoassays cross-react with *trans*-3′-hydroxycotinine, but not with nicotine or nornicotine [82].

Moreover, determination via chromatographic separation by either LC or GC has been largely preferred due to their high sensitivity and selectivity. Additionally, chromatographic analysis grants the simultaneous determination of nicotine and all metabolites, becoming quicker and more efficient [6].

In the last years, the coupling of LC with MS has been the most adopted for the purpose. The most reported mode of ionization was electrospray ionization (ESI) in the positive mode, although atmospheric pressure chemical ionization (APCI) is usually less susceptible to ion suppression [84].The ESI mode was chosen according to analytes polarity, size and presence of heteroatoms and functional groups. Although positive mode is commonly preferred, the negative mode can be a better option due to its improved sensitivity (ionization efficiency) and lower detection limits, hence adopted by some authors [108].

Kataoka et al. [98] presented a LC-MS method to determine nicotine, cotinine, and related alkaloids (nornicotine, anabasine, and anatabine) in urine and oral fluid samples. In this work, LODs of 15 to 40 pg/mL were reported [98]. Although LC-MS is sensitive and accurate enough, the most widely used analytical instrumentation for these target analytes is LC-MS/MS. The latter is acknowledged for being faster, more sensitive, and specific, permitting the determination of diminutive levels of the compounds in tobacco smoke exposure.

McGuffey et al. [84] developed a rapid and low-cost method of LC-MS/MS for the simultaneous determination of nicotine, six metabolites, and two minor tobacco alkaloids in smokers’ urine samples. The authors used ESI mode due to one of the analytes (nicotine 19-*N*-oxide) being thermo labile, hence quantification by APCI would not be possible [84]. This multi target method resulted in LODs ranging from 0.41 to 3.53 ng/mL, which can be considered quite sensitive. APCI ionization was adopted by Gray et al. [82], when a LC-MS/MS method was developed to determine nicotine and four metabolites in meconium from both tobacco-exposed and nonexposed neonates. The authors reported LOQs between 1.25 and 5 ng/g [82].

Pérez-Ortuño et al. [89] proposed the simultaneous quantification of nicotine and cotinine in multiple biological matrices (urine, oral fluid, and hair) using hydrophilic interaction (HILIC) ultra-high performance liquid chromatography-tandem mass spectrometry (UHPLC-MS/MS) [89]. This ultrafast method allowed the reduction of chromatographic time down to 2 min, and its sensitivity resulted in LOQs between 0.0020 and 0.026 ng/mg for hair and 0.040 and 0.48 ng/mL for urine and oral fluid specimens. Great chromatographic resolution was obtained under HILIC conditions, since cotinine, and particularly nicotine, are polar compounds [89]. Later on, Feng et al. [80] reported a method for the same analytes in oral fluid, but with reverse-phase LC-MS/MS, resulting in LOQS between 1 and 2 ng/mL. A reverse phase stationary phase was also used in Inukai et al. [95] work that aimed the determination of nicotine and cotinine in hair samples by LC-MS/MS. The LOQs of nicotine and cotinine in hair were 0.0075 and 0.0044 ng/mg, respectively, which can be considered extremely sensitive considering that the authors used only 1 to 2 mg of sample. The HILIC-UHPLC-MS/MS approach of Pérez-Ortuño et al. [89] used 10 mg of hair. A new type of ionization source, iFunnel ionization source, adopted for LC-MS/MS is reported one year later by Pérez-Ortuño et al. [88] to quantify nicotine, cotinine, NNN, NNK and NNAL in hair samples. Commonly, far less than 1% of analyte ions produced by ESI enter the mass spectrometer. The iFunnel ionization source gathers the high ESI ion generation and the focusing of Agilent Jet Stream with a hexabore capillary sampling array, enabling a much greater fraction of the ESI spray plume to enter the mass spectrometer [109]. This, consequently, enhanced the sensitivity in LC-MS/MS, and allowed Pérez-Ortuño et al. to obtain LOQs of 25, 2.5, 0.25, 0.10, and 0.063 pg/mg for nicotine, cotinine, NNN, NNK and NNAL, respectively, with 20 mg of sample [88].

Although LC-MS/MS technique is the most adopted for these target analytes determination in the last years, GC-MS methods have also been successfully applied. Toraño et al. [96] used the latter to determine thiocyanate, nicotine, and cotinine in human urine, saliva, reporting LOQs of 1 µg/mL for thiocyanate and 10 ng/mL for nicotine and cotinine. Furthermore, Kim et al. [91] used a GC-MS method to determine nicotine, cotinine, norcotinine, and *trans*-3′-hydroxycotinine in oral fluid obtaining a LOQ of 5 ng/mL for all compounds. These limits are commonly greater than those reported with LC-MS methods; hence, GC-MS might not be the most sensitive option, but still efficient for the purpose. In order to improve these GC methods’ sensitivity, MS/MS detection can be coupled, and the analytical method proposed by da Fonseca et al. [100] is the proof of that. These authors determined nicotine, cotinine, and *trans*-3′-hydroxycotinine in 200 µL of oral fluid by GC–MS/MS reaching LOQs of 0.5 ng/mL for all biomarkers.

Moreover, and nowadays, high-resolution accurate mass spectrometry (HRAM-MS) detection together with non-targeted screening (NTS) is considered the key methodology to characterize the chemical composition of many biological specimens, even the most complex [110]. An example of that was the method developed by Carrizo et al. [104] that allowed the determination of twelve TS exposure biomarkers, including glucuronides, in 300 µL of urine and saliva. The authors report a single run analysis using atmospheric pressure solid analysis probe (ASAP) coupled to HRAM-MS, namely high resolution mass spectrometry with quadrupole and time of flight detector (Q-TOF-MS). The greatest advantage of this method is the elimination of the sample clean-up steps, commonly adopted before the instrumental analysis. Nevertheless, the authors conclude that even though ASAP-Q-TOF-MS is an attractive, fast, and useful technique, it will result in greater LOQs than an LC-MS/MS, since the direct analysis of the biological specimen will have the interferences of proteins, fat matter and metabolites not removed by previous extraction techniques [104].

## 6. Conclusions

There is no doubt that seeking for new biomarkers for ETS exposure, particularly if one takes into account the new and emerging tobacco products, is a challenging research trend nowadays. Many of these products are composed only of nicotine, and this limits the number of candidate biomarkers. In fact, at the moment there is no study that differentiates exposure to tobacco smoke from exposure to electronic cigarette products.

The better knowledge of metabolic profiles of the involved compounds is an excellent tool aiming at finding the best substance to function as biomarker of exposure. Unfortunately, no biomarker by itself is capable of differentiating the different types of tobacco consumers. In addition, many biomarkers of exposure, despite of allowing distinguishing between smokers and non-smokers, do not provide comprehensive data as regards dose-response and its correlation to intensity and frequency of usage or to consumption cessation or reduction.

The need of using metabolites of the compounds present in tobacco was demonstrated in the case of exposure to tobacco smoke, and from these cotinine was among the most used; however, other biomarkers were also tested in order to understand whether treatments for tobacco dependence were working and the individual did actually quit smoking. Oral fluid and urine were the most used specimens to assess exposure due to the high levels of cotinine present in this type of sample. In addition, the possibility of using hair samples has been widely reported in recent years, but there is lack of consensus as regards washing procedures to eliminate external contamination; this is important to differentiate active from passive smokers, and as such further studies are necessary on this.

Concerning sample preparation, it is expected that analysts move towards more environmentally friendly procedures, reducing laboratorial waste. In addition, the implementation of automated methods for compound extraction may solve several problems in terms of reproducibility.

Searching for correlations between the different biological specimens, as well as establishing adequate cut-off values, will be one of the future fields of research. For this, sophisticated and sensitive analytical instrumentation will be needed, for instance time-of-flight or orbitrap technologies, allowing detecting lower concentrations of the compounds. Furthermore, the application of these techniques to metabolomics may allow identifying adequate candidate biomarkers.

## Figures and Tables

**Table 1:** Sample preparation and analytical methods to determine biomarkers of exposure to environmental tobacco smoke (ETS) in biological specimens.

Compound	Sample (Amount)	Sample Preparation	Analytical Technique	LOD, ng/mL (pg/mg of Hair)	LOQ, ng/mL (pg/mg of Hair)	Recovery (%)	Reference
**Nicotine and tobacco alkaloids**							
Nicotine, Cotinine	Hair: 20 mg	Incubation: 1 mL 1 M NaOH, 2 M KCl aqueous solution (30 min at 80 °C); LLE (5 mL dichloromethane and 5 mL dichloromethane:isopropanol (75:25))	LC-MS/MS (iFunnel ionization +)	-	0.10–25	-	[88]
Nicotine, Cotinine	Hair: 10 mg	Incubation: IS solution (80 °C, 30 min); LLE: 0.5 mL of dichloromethane	LC-MS/MS (ESI+)	0.66–8.6	2–26	>90	[89]
	Urine and Oral fluid: 0.5 mL	LLE: 0.5 mL of dichloromethane		0.0132–0.158	0.04–0.48		
Cotinine Oxide, Nicotine 1′ Oxide, *trans*-3′-hydroxycotinine, Norcotinine, Cotinine, Nornicotine, Anatabine, Anabasine, Nicotine	Urine: 0.1 mL	Enzymatic hydrolisis: 160 μL of β-Glucuronidase solution (for free samples); PP: 0.5 mL of cold acetone	LC-MS/MS (ESI+)	0.41–3.53	-	76–99	[84]
Nicotine, Cotinine	Oral fluid: 0.5 mL	SPE: Phenomenex Trace B	LC-MS/MS (n.s.)	-	1–2	-	[80]
Nicotine	Oral fluid: 0.25 mL	LLME: chloroform	GC-MS (EI)	-	-	-	[90]
			IONSCAN^®^-LS IMS (⁶³Ni foil radioactive ionization source)	9	-	99	
Nicotine, Cotinine, *trans*-3′-hidroxicotinine	Oral Fluid and Plasma: 0.5 mL	LLE: 2 mL of chloroform/isopropyl alcohol	LC-MS/MS (ESI)	-	-	-	[85]
Nicotine, Cotinine, Norcotinine, *trans*-3′-hidroxicotinine	Oral Fluid: 0.5 mL	SPE: Clean Screen^®^ ZSDAU020, 200 mg–10 mL	GC/MS (EI)	5	5	67–117.8	[91]
Nicotine, Cotinine, Norcotinine, *trans*-3′-hidroxicotinine	Oral Fluid: 0.5 mL	SPE: Clean Screen^®^ ZSDAU020	LC-MS/MS (ESI+)	-	0.2–1	63.6–101.2	[92]
Nicotine, Cotinine, *trans*-3′-hidroxicotinine, Nornicotine, Norcotinine	Meconium: 500 mg	Enzymatic hydrolysis (for total concentration): 0.1 M β-Glucoronidase potassium phosphate buffer; SPE: Clean Screen^®^ ZSDAU020	LC-MS/MS (APCI+)	1.25–5	1.25–5	56.2–95.7	[93]
Nicotine, Cotinine, *trans*-3′-hidroxicotinine	Meconium: 250 mg	Hydrolysis with 3 M KOH for 30 min at 60 °C, addition of 500 μL 1 M HCl. SPE: Oasis^®^MCX	LC-MS/MS (ESI+)	2–10	2–10	73.2–125.4	[94]
Nicotine, Cotinine	Hair: 1–2 mg	Incubation: distilled water (80 °C, 30 min); in-tube SPME: Carboxen 1006 PLOT capillary column (60 cm × 0.32 mm i.d.)	LC-MS/MS (ESI+)	0.13–0.45 (pg/mL)	4.4–7.5	87–96.1	[95]
Nicotine, Cotinine	Hair: 20 mg	Incubation: 800 μL of water (10 min, 100 °C); LLE: 100 μL of dichloromethane	GC-MS (n.s.)	-	-	≈80–90	[96]
	Urine: 0.8 mL	LLE: 150 μL of alkylating solution (HTAB+ PFBBr in methanol: dichloromethane (1:2, *v*/*v*)) and 100 µL of a saturated sodium chloride solution		0.06–0.6	-		
	Oral fluid: 0.8 mL	LLE: 100 μL of dichloromethane and 100 µL of a saturated sodium chloride solution		0.6	-		
Nicotine-*N*-β-*D*-Glucuronide, Cotinine-*N*-Oxide, *trans*-3′-hydroxycotinine, Norcotinine, *trans*-Nicotine-1′-oxide, Cotinine, Nornicotine, Nicotine, Anatabine, Anabasine and Cotinine-*N*-β-*D*-Glucuronide	Urine: 1 mL	Acidification: 1.5 mL of 5 mM aqueous ammonium formate (pH 2.5); SPE: combination of Oasis^®^ HLB and Oasis^®^ MCX cartridges	LC-MS/MS (ESI+)	1–25	1–50	52–88	[12]
	Plasma: 1 mL	PP: 1 mL of 10% aqueous trichloroacetic acid; SPE: combination of Oasis^®^ HLB and Oasis^®^ MCX cartridges		0.25–25	1–50	51–118	
Nicotine-*N*-β-*D*-Glucuronide, Cotinine-*N*-Oxide, *trans*-3′-Hydroxycotinine, Norcotinine, *trans*-Nicotine-1′-oxide, Cotinine, Nornicotine, Nicotine, Anatabine, Anabasine and Cotinine-*N*-β-*D*-Glucuronide	Hair: 20 mg	Incubation: 1 mL of 1 M sodium hydroxide solution (1 h, room temperature); SPE: Oasis^®^ MCX	LC-MS/MS (ESI+)	0.1–0.10	0.5–0.10	13.5–117.8	[83]
Cotinine	Blood: 5 drops (finger-prick DBS) or 0.05 mL (reconstitued DBS)	DBS: 100 μL methanol	LC-MS/MS (ESI+)	-	0.25	-	[86]
Cotinine	Oral fluid: 1 mL	PP: 10 μL acetonitrile (4 °C, 20 min); μSPE-BI-LOV: OASIS^®^ HLB cartidges	HPLC-DAD	1.5	3	95.9	[97]
Nicotine, Cotinine, Nornicotine, Anabasine, and Anatabine	Oral fluid: 0.2 mL	In-tube SPME: CP-Pora PLOT amine capillary column (60 cm × 0.32 mm i.d., 10 µm film thickness)	LC-MS (ESI+)	0.015–0.040	-	83–98.2	[98]
	Urine: 0.1 mL					83.2–97.4	
Cotinine	Urine: 0.02 mL	Automated on-line SPE: Extraction column (Inertsil ODS-3 33 mm × 4.6 mm, 5 μm) and 10-port switching valve (two-position microelectric actuator from Valco Instrument Co., Ltd. Houston, TX, USA)	LC-MS/MS (ESI+)	0.005	0.02	-	[99]
Cotinine-*N*-glucuronide, Nicotine-*N*-glucuronide, *trans*-3′-hydroxycotinine-*O*-glucuronide, *trans*-3′-Hydroxycotinine, Cotinine, Nicotine	Liver and Placenta: 0.25 g	SLE: Isolute-supported liquid extraction columns	LC-MS/MS (ESI+)	0.7–7 ng/mg	1–10 ng/mg	31.3–107	[15]
Nicotine, Cotinine, *trans*-3′-hydroxycotinine	Oral fluid: 0.2 mL	SPE: Oasis^®^ MCX cartidges	GC-MS/MS (EI+)	0.5	0.5	84.6–99.8	[100]
**Tobacco-specific *N*-nitrosamines (TSNA)**							
NNAL, NNAL-*O*-Gluc, and NNAL-*N*-Gluc	Urine: 0.08 mL	True Paper 96-well plates with PBS followed by Isolute SLE+ diatomaceous earth solid-phase extraction 96-well plates and Oasis^®^ MCX SPE cartidges	LC-MS/MS (ESI+)	-	-	-	[35]
	Urine: 0.06 mL	True Paper 96-well plates with alcaline hydrolsis (0.5 M sodium hydroxide) followed by Isolute SLE^+^ diatomaceous earth solid-phase extraction 96-well plates and Oasis^®^ MCX SPE cartidges		-	-	-	
	Urine: 0.04 mL	True Paper 96-well plates with enzymatic hydrolsis (β-Glucoronidase) followed by Isolute SLE^+^ diatomaceous earth solid-phase extraction 96-well plates and Oasis^®^ MCX SPE cartidges		-	-	-	
NNN	Toenails: 40–100 mg	Incubation: 2 mL 1 N sodium hydroxide (50 °C, overnight); SPE: Chem Elut and Oasis^®^ MCX; NPE: Bond-Elut Silica cartridges	LC-MS/MS (ESI+)	-	-	-	[87]
NNN, NNK, NNAL	Hair: 20 mg	Incubation: 1 mL 1 M NaOH, 2 M KCl aqueous solution (30 min at 80 °C); LLE (5 mL dichloromethane and 5 mL dichloromethane:isopropanol (75:25))	LC-MS/MS (iFunnel ionization +)	-	0.10–25	-	[88]
NNAL, NNN, NNK, NAB, NAT	Urine: 5 mL	Total TSNA procedure: Enzymatic hydrolisis (0.5 mL β-Glucoronidase); SPE (Chem Elut); MIP’s: NNAL MIP	LC-MS/MS (ESI+)	0.00004–0.01	-	53–67	[101]
		Free TSNA procedure: SPE (Chem Elut); MIP’s: NNAL MIP					
**Thiocyanate**							
Thiocyanate	Nasal fluid: ≈0.5 g	Direct injection	Ion chromatography (anionic)	0.02	-	-	[79]
Thiocyanate	Hair: 20 mg	Incubation: 800 μL of water (10 min, 100 °C); LLE: 100 μL of dichloromethane	GC-MS (n.s.)	-	<0.5	≈80 to 90	[96]
	Urine: 0.8 mL	LLE: 150 μL of alkylating solution (HTAB+ PFBBr in methanol: dichloromethane (1:2, *v*/*v*)) and 100 µL of a saturated sodium chloride solution		0.06	1.0		
	Oral fluid: 0.8 mL	LLE: 100 μL of dichloromethane and 100 µL of a saturated sodium chloride solution		-	<0.5		
Thiocyanate	Placenta: 5 g	ASE with water; diatomaceous earth mixture	Ion chromatography (anionic)	0.01	-	92.7	[102]
Thiocyanate	Semen: n.s.	Direct injection	Ion chromatography (anionic)	0.01	0.03	-	[78]
**Polycyclic aromatic hydrocarbons (PAHs)**							
Naphthalene, Acenaphthylene, Acenaphthene, Fluorene, Phenanthrene, Anthracene, Fluoranthene, Pyrene	Oral fluid: 0.5 mL	HS-SPME: (PDMS- 100 µm)	GC-MS/MS (EI+)	0.0007–0.0222	0.0008–0.0264	-	[103]
1-hydroxypyrene, 9-hydroxyphenanthrene, Muconic acid, 3-Hydroxypropylmercapturic acid	Urine and Oral fluid: 0.3 mL	Direct injection	ASAP-Q-TOF-MS (API+)	100–500 (for 1-hydroxypyrene, 9-hydroxyphenanthrene)	1000–3000 (for 1-hydroxypyrene, 9-hydroxyphenanthrene)	-	[104]
9-hydroxyphenanthrene, 1-hydroxypyrene		LLE: 1 mL of chloroform-methanol (1:2, *v*/*v*)	UHPLC-MS/MS (APCI+)	10–50	100–500	-	
PheT	Urine: 0.1 mL	V-bottomed 96-well collection plates with enzymatic hydrolisis (β-Glucoronidase and arylsulfatase) followed by Strata SDB-L SPE	GC-MS/MS (NICI)	-	-	-	[35]

Legend: ^+^: positive ionization mode; APCI: atmospheric-pressure chemical ionization; API: atmospheric pressure ionization; ASAP-Q-TOF-MS: atmospheric solids analysis probe quadrupole time of flight mass spectrometry; ASE: accelerated solvent extraction; DBS: dried blood spots; EI: electron ionization; ESI: electrospray ionization; GC-MS: gas chromatography MS; GC-MS/MS: GC tandem mass spectrometry; HPLC-DAD: high performance liquid chromatography with diode-array detector; HS-SPME: headspace solid phase microextraction; IONSCAN^®^-LS IMS: IONSCAN LS ion mass spectrometry; LC-MS: liquid chromatography mass spectrometry; LC-MS/MS: LC tandem mass spectrometry; LLE: liquid-liquid extraction; MIP: molecular imprinted polymer; NICI: negative ion chemical ionization; NPE: normal phase extraction; n.s.: non specified; PP: protein precipitation; SLE: supported liquid extraction; SPE: solid phase extraction; SPME: solid phase microextraction; UHPLC-MS/MS: ultra-high-performance liquid chromatography; μ-SPE-BI-LOV: microSPE bead injection lab-on-valve.

## Data Availability

Data sharing is not applicable to this article.
